# The Bandage in Its Application to Fractures, &c.

**Published:** 1850-01

**Authors:** 


THE BANDAGE IN ITS APPLICATION TO FRACTURES, ETC.

In our notice of Dr. Stone we promised an essay on the bandage, and it was prepared for this number, but we have been compelled to postpone its publication until February.

